# Salivary Calculus

**Published:** 1856-06

**Authors:** F. D. Thurman

**Affiliations:** Dentist, Atlanta


### ARTICLE III.

*Salivary Calculus.* By F. D. THURMAN, Dentist, Atlanta.

From some cause or other, it seems that medical students and physicians pay comparatively little attention to the structure of the teeth, and diseases of the adjacent parts. I suppose this is owing to the fact, that the diseases of these parts belong more to the speciality of the dental surgeon than to general medicine; and consequently (as they are seldom called on to treat diseases of the teeth and mouth,) they are not willing to devote much time to the study of these subjects. This apology, to some extent, is admissible; but when we take into consideration, the numerous diseases (in acute and chronic form,) growing out of the existing causes, located in these parts, it certainly seems to me that physicians should at least be prepared to advise their patients, in reference to a proper course of treatment. But as a proof that they are not always prepared to do this, I will simply relate a case in point.

A patient, aged about twenty-three years, of scorbutic habit, called on me, and asked my advice in reference to a diseased condition of his gums, which he informed me had been sore and painful for several years, and growing worse continually. Upon examination, I discovered that his teeth were literally coated with tartar, or salivary calculus, particularly the buccal surfaces of the superior molars, where it was about one-eighth of an inch thick, and had insinuated itself between the gums and necks of the teeth, causing a general inflammation of the parts, and gradual absorption of the alveolar process. The teeth, in consequence, being so loose that they might be removed with the fingers. The periosteal membrane of nearly all of the teeth, was in an inflamed condition, gums spongy, and morbidly sensitive, secretions of the mouth vitiated, and acting rapidly on the teeth, most of which were decayed. And it is useless to add, that his breath (exhaled through this morbid mass) was extremely disagreeable. The following conversation ensued. "The whole of your trouble," said I, "is occasioned by the accumulation of tartar, which has excited inflammation in the gums, and the membranes covering the roots of the teeth. I am sorry you did not call sooner, as it will be impossible to make your teeth firm in their sockets again, since a considerable portion of the bone that surrounded their necks has been destroyed by the disease. Though, by the use of astringent and stimulating washes, which I shall prescribe for you, the gums in a short time will be rendered healthy and firm, and the teeth, in consequence of a healthy condition of these parts, will not be quite as loose as they are at present. But in the first place, I must remove the cause, which, however, I will defer for another sitting, as it is growing late."

"What, Doctor, you don't mean to take the tartar off of my teeth, do you?"

"Certainly, I do."

"Oh, no, you can't do that, because our family physician says it must not be taken off, that it is an advantage to the teeth, and should never be removed."

"Did he tell you in what way the teeth are benefitted by it?"

"Yes, he said it kept them from aching and decaying."

"Did he say this after examining and seeing the condition of your mouth and gums?"

"Yes, after he had examined them frequently."

"Did he prescribe for the disease of your gums?"

"Yes, he has been treating them for nearly three years; sometimes they appear to be better, and then worse again, and now they are getting worse all the time."

"Yes, no doubt of it, and unless the tartar is removed, they will continue to grow worse until the teeth are lost. If you will submit to proper treatment, you may be relieved in a very short time. But if I am not to have entire control of the treatment, I cannot undertake it at all."

"Well, I don't know what to do,—our doctor says, that tartar is of no disadvantage to the teeth, and as a proof of the faith of his opinion, he never has it removed from his own teeth. Though I will see him again, and call and let you know my conclusion."

The next afternoon, the young man called and informed me that the doctor wished me to take his case in hand, and do the best I could for him. I did so, and in about four weeks from that time, the doctor called and had the tartar removed from his own teeth; requesting me at the same time to be certain not to leave the smallest particle for it to rebuild on. I will just say here, that if the reader imagines this physician to be wanting in intellectual power, or deficient in general information in medical science, he is very much mistaken. He was a graduate of one of our first class medical colleges, and deservedly enjoyed a considerable reputation as physician and surgeon. But, like most other physicians, he paid comparatively little attention to diseases that he did not expect to be called on to treat; which, as a general rule, I think a very good one, as it is important that every physician and surgeon should be thoroughly acquainted with the proper treatment of diseases pertaining to his speciality, and the time allotted for man to live is too short for him to become perfect, in all the various branches of medicine, surgery, &c. But as the general system sympathises so decidedly with diseases even of the most remote character, I must say, that surgeons should be prepared to say when their patients should be submitted to medical treatment, and physicians should be able to decide correctly, as to the proper time to call in the surgeon, &c. But to the subject.

Tartar, or salivary calculus, may be detected on the teeth, in a greater or less degree, of every individual, who fails to attend properly to the cleansing of these organs. It is divided, by writers on the subject, into many varieties, five of which I think sufficient for all practical purposes, to wit: the black, the dark brown, the yellow brown, the white, and the dark green.

The variations in color, as well as consistency, is owing to a difference in the proportion ot their chemical constituents; the black is the hardest, the white the softest, and the intermediate colors are hard or soft, as they approach the black or white.

*Its chemical constituents.*—Tartar, or salivary calculus, is composed of earthy and animal matter, the former generally predominating, but as two specimens of the same consistency are seldom met with, of course a chemical analysis by different experimenters or by the same experimenter at different times, will disagree, as may be seen by the following analysis:

*Analysis by Dr. Pepys for Dr. Tox.*

| | |
|---|---|
| Phosphate of lime, | 70 |
| Fibrina, or cartilage, | 18 |
| Animal fat or oil, | 6 |
| Loss, | 6 |
| | 100 |

*Two analysis by Dr. Wright.*

| | First. | Second. |
|---|---|---|
| Carbonate of lime, | 81.3 | 79.4 |
| Phosphate of lime, | 4.1 | 5.0 |
| Animal matter and mucus, | 7.1 | 9.5 |
| Potash, soda, &c., | 6.2 | 1.3 |
| Loss, | 1.3 | 4.8 |
| | 100.0 | 100.0 |

*Analysis by Dr. Dwinelle, a scientific dentist of Cazenovia, N. Y.*

| | |
|---|---|
| Phosphate of lime, | 60 |
| Carbonate of lime, | 14 |
| Animal matter and mucus, | 16 |
| Water and loss, | 10 |
| | 100 |

*Analysis by Berzelius.*

| | |
|---|---|
| Phosphate of lime and magnesia, | 79.0 |
| Salivary, mucus and salivine, | 13.5 |
| Animal matter, | 7.5 |
| | 100.0 |

The dark, or hard varieties, contain more earthy and less animal matter, whilst the white, or soft kinds, contain a larger proportion of animal matter.

*Its origin and manner of formation.*—As is the case on most other subjects, theories with different degrees of plausibility have been introduced in relation to the origin of this substance. Several French writers have introduced theories peculiar with themselves. Jourdain seemed, at one time, to be sanguine that it was secreted by glands scattered over the periosteum of the teeth. Gariot says it comes from the gums, but Serres, more unreasonable than either, professes to have discovered glands, whose peculiar function it is to secrete this filth. But nearly all writers of a recent date agree in ascribing its production to the saliva, and three very strong proofs of the fact are: 1st. That the elements of its composition have, by several chemical analysis, been detected in the saliva itself. 2d. It invariably commences its deposit on the buccal surfaces of the inferior incisors just opposite the ducts of the parotid and sublingual glands; and 3dly. It is occasionally formed in the very channels of the ducts themselves. It usually commences in the form of dirty whitish deposite mixed with mucus, and soon hardens. The longer it remains the harder it gets, until its under surface becomes almost as hard as the ivory of the teeth to which it adheres; the exterior surface being softer in proportion to its more recent deposit. It seldom accumulates to any extent on the teeth of children and very young persons. I have occasionally seen the teeth of boys, at the age of sixteen or eighteen, almost entirely covered with it; but as a general rule, it does not accumulate much until after the age of twenty or twenty-five, and the old are more subject to it than the middle aged; nervous temperaments are more subject to it than sanguine, and bilious more than nervous. It accumulates in but small quantities on the teeth of those who are possessed of the best constitutions, and with this class it is generally confined in its limits to the necks of the inferior incisors.

*Its effects.*—With the exception of caries, nothing is so destructive to the teeth as tartar, to say nothing of the disgusting effect its presence produces on the optics and olfactories of friends and associates. In its hardened state, it has no direct chemical effect on the teeth, but indirectly, by getting up a chronic inflammation of the gums and vitiating the secretions of the mouth, it becomes a very potent agent in the decomposition of these organs; but not seeming to be satisfied with this mode of attack, it insinuates itself between the necks of the teeth and gums, destroys the attachment, loosens the teeth, and so diseases the gums as to cause them to secrete fetid matter, irritates and excites inflammation of the periosteum, and the wasting and final destruction of the alveolar process, and the consequent loss of the teeth. The teeth of old people, though they may have withstood an attack from caries, generally fall victims to this substance, and not one in a hundred can tell why it is that their teeth are gradually loosening and dropping out.

The effects of tartar are not confined to the diseases mentioned, but it not unfrequently produces indolent ulcers and tumors of the gums, necrosis and exfoliation of large portions of the maxillary bones, and occasionally inflammation and altered conditions of the secretions of the mucus membrane of the antrum, where it is liable, particularly in debilitated or diseased habits of body, to produce any of the formidable diseases consequent upon a disturbance of the interior structure of this cavity, such as purulent condition of the secretions, engorgement of the cavity, tumors and ulcers of various kinds, mollities ossium, &c. &c.

*Treatment.*—Notwithstanding this is a dirty substance, the operator should be prepared with clean napkins, and bright instruments, for its removal. The instruments should be of various shapes and sizes, so that any point may be reached without injuring the gums.

The patient being comfortably seated in a suitable light, the operator, after washing his hands, and placing his instruments and napkins in a convenient position, should commence with a gentle, careful, but firm hand, and remove piece after piece until every particle is removed, and the teeth are clean and nice. If the smallest particle is left, even though it may not be seen without the assistance of a microscope, it will form a nucleus around which new deposits will soon accumulate. The surfaces should now be polished with a fine powder on the points of little slips of wood, shaped for the purpose, and then finished, by rubbing them well with floss silk.

The patient should now be directed to use great precaution in keeping them clean. Tartar is more liable to collect on teeth that have once been the subjects of it, owing to the fact, that the margins of the gums never adhere as firmly to them after being once detached, and consequently the chances for its finding a location for the re-commencement of its deposit where it is not likely to be worked away by the fluids of the mouth are greatly increased. And in addition to this, the gums having receded more or less, on account of its former presence, and exposed the cementum, which, in consequence of its not being as smooth as the enamel, forms a more suitable surface for its firm attachment, but by using a brush (diped in a suitable dentrifice,) freely, several times a day, its reaccumulation may be prevented. The powders forming the dentrifice should be impalpable. If the least particle of grit can be detected in grinding it between the teeth, it is not fit for constant use. But powders put up by apothecaries and druggists for dental purposes, injure the teeth oftener by their chemical than mechanical properties. The practice of mixing mineral acid with powders for the purpose of whitening the teeth, cannot be too highly deprecated. The teeth, in this way, are soon made white, but it is at the expense of a portion of their enamel, the surface of which soon looses its lustre and polish and becomes more or less roughened, and if the use of such powder is persisted in, the teeth are soon destroyed. The patient should also be directed to attend to the approximal surfaces of the teeth, and as their surfaces cannot be reached with the brush, suitable picks and threads of floss silk should be recommended.

